# Antiliver Fibrosis Formula of Fuzheng Huayu Alleviates Inflammatory Response

**DOI:** 10.1155/2022/5752803

**Published:** 2022-11-12

**Authors:** Qing-Qi Chang, Yi-Feng Pan, Jia-Yi Yang, Rong-Sheng Li, Chun-Lu Yuan, Ya-Fang Liao, Dan-Dan Zhang, Cheng-Hai Liu

**Affiliations:** ^1^Institute of Interdisciplinary Integrative Medicine Research, Shanghai University of Traditional Chinese Medicine, Shanghai 201203, China; ^2^Shanghai Huanghai Pharmaceutical Co. Ltd., Shanghai 200051, China; ^3^Institute of Liver Diseases, Shuguang Hospital Affiliated to Shanghai University of Traditional Chinese Medicine, Shanghai 201203, China

## Abstract

Fuzheng Huayu's (FZHY) formula ameliorated liver fibrosis in clinical and experimental practice. Based on the close link between fibrosis and inflammation, its anti-inflammatory effect and related mechanisms were explored in this present study. With the aid of the inflammatory macrophage model, FZHY significantly blocked nitrite accumulation without observable cytotoxicity due to its suppression of inducible nitric oxide synthase (iNOS) gene and protein expressions in a concentration-depended manner. Proinflammatory mediators including IL-6, CD86, and CD40 were also restrained by FZHY. Interestingly, FZHY induced anti-inflammatory mediators heme oxygenase 1 (HO-1) and peroxisome proliferator-activated receptor *γ* (PPAR-*γ*) expressions simultaneously. Downregulation of iNOS and miR-155 and upregulation of PPAR-*γ* were also observed in CCl_4_-induced liver fibrosis mice upon FZHY administration. Mechanically, FZHY strikingly eliminated the phosphorylation of STAT1 and MAPK. Taken together, FZYH regulated the balance of proinflammatory and anti-inflammatory mediators partially via modulating STAT1/MAPK pathways and the miR-155/PPAR-*γ* axis.

## 1. Introduction

Hepatic fibrosis is a chronic wound-healing response characterized by inflammation [1]. Genetic changes, hepatitis virus infections, excessive alcohol consumption, lipid metabolic disorders, and autoimmune diseases trigger continuous liver injury and subsequent chronic inflammation that lead to liver fibrosis [2, 3]. Understanding the underlined inflammatory mechanisms is critical to developing strategies to control fibrosis [4].

Hepatic macrophages are the primary immune cells, consisting of liver-resident macrophages, monocyte-derived macrophages, and Kupffer cells that trigger liver inflammatory response and also play a crucial role in the pathologic progress of fibrosis [5]. Traditionally, macrophages can be polarized into proinflammatory/M1 macrophages or anti-inflammatory/M2 macrophages upon different microenvironmental stimulations. M1 macrophages predominantly express inducible nitric oxide synthase (iNOS) and secrete classical proinflammatory cytokines such as interleukin (IL)-6 and IL-1β to exaggerate the inflammatory response induced by *T* helper (Th)-1 signals such as lipopolysaccharide (LPS) and interferon (IFN)-*γ*. Accumulating evidence strongly implies that iNOS-derived nitric oxide (NO) has been associated with the pathogenesis of liver diseases during inflammatory conditions. These inflammatory mediators also emerge as vital profibrotic hubs. IL-1*β* promotes liver fibrosis partially in an IL-17-dependent manner [6]. Thus, targeting macrophages and inhibition of M1 macrophage-led inflammation development are approaches to interfere with fibrosis [7].

MicroRNAs (miRNAs) are small, noncoding RNAs that negatively control target gene expressions by promoting degradation or translational inhibition. miR-155 exerts a proinflammatory effect during the progress of hepatic fibrosis in immune cells [8]. PPAR-*γ* is involved in the progression of liver fibrosis as one of the target genes of miR-155 [9]. CCl_4_ administration decreases the expression of PPAR-*γ* in liver tissue and the antifibrotic effect of crocin partly via enhancing PPAR-*γ* to mediate inflammatory response and fibrogenic events [10].

Fuzheng Huayu's formula (FZHY) was approved by the Chinese State Food and Drug Administration (SFDA) (No : Z20050546) as an antifibrotic medicine in 2002. FZHY is composed of six Chinese medicines, including *Radix Salvia Miltiorrhizae* (Danshen)*, Cordyceps* (Chong Cao), *Semen Persicae* (Taoren), *Pollen Pini* (Song Huafen), *Gynostemma Pentaphyllammak* (Jiaogulan), and *Fructus Schisandrae chinensis* (Wuweizi), which is used to invigorate blood circulation, remove blood stasis, tonify essence, and nourish liver according to the theory of TCM. FZYH's antifibrotic effect has been confirmed in accumulating experimental and clinical evidence [11–20]. Current data showed FZHY alleviated inflammatory cytokines TNF-*α* and IL-6 and profibrotic genes VEGF and TGF-*β*1 in the liver fibrotic rat model [21]. However, its anti-inflammatory mechanisms remain to be elucidated.

Thus, in this present study, we evaluated the anti-inflammatory effect and relevant mechanisms of FZHY.

## 2. Materials and Methods

### 2.1. Materials

FZHY was obtained from Huanghai Pharmaceutical Co. (Shanghai, China). RPMI 1640 medium, fetal bovine serum (FBS), and 0.25% trypsin were purchased from Gibco (Rockville, MD, USA). BCA protein assay kit and TRIzol reagent were purchased from Invitrogen (Carlsbad, CA, USA). Special antibodies including p-JNK(#9251; 1 : 1000), T-JNK(#9252; 1 : 1000), p-ERK(#9101; 1 : 1000), T-ERK(#9102; 1 : 1000), p-P38(#9211; 1 : 1000), T-P38(#9212; 1 : 1000), p-STAT-1(#7649; 1 : 1000), T-STAT-1(#14994; 1 : 1000), and iNOS(#13120; 1 : 1000) were purchased from cell signaling technology (Beverly, MA, USA). Anti-HO-1 (ab68477; 1 : 10000), CD40 (ab252428; 1 : 1000), IL-6 (ab259341; 1 : 1000), and horseradish peroxidase-labeled goat antirabbit IgG were purchased from Abcam (Cambridge, UK). CD86 (13395-1-AP; 1 : 500) was purchased from ProteinTech Group (Chicago, IL, USA). Polyvinylidene difluoride (PVDF) membranes, western blotting detection reagent ECL, and murine recombinant IFN-*γ* were purchased from Millipore (Bedford, MA, USA). LPS, 1400W, Griess reagent, and MTT were purchased from Sigma-Aldrich (St. Louis, MO, USA). MicroRNA primers, transcription kits, and universal PCR master mix were obtained from GenePharma Company (Shanghai, China). Standard compounds sodium danshensu, salvianolic acid B, and adenosine were purchased from the National Institutes for Food and Drug Control (Beijing, China).

### 2.2. High-Performance Liquid Chromatography (HPLC) Assay

FZHY standardization was performed using HPLC fingerprinting with chemical standard compounds such as sodium danshensu, salvianolic acid B (two compounds isolated from *Radix Salvia Miltiorrhizae*), and adenosine (a compound isolated from Cordyceps) according to Chinese Pharmacopoeia (2015 edition).

### 2.3. Cell Culture

RAW 264.7 cells from the American Type Culture Collection (ATTC, Rockville, MD) were cultured in RPMI 1640 medium supplemented with 10% FBS. The cell passages below 10 were used, and three replicates were performed in each experiment.

### 2.4. Cell Viability

The effect of FZHY on the proliferation of RAW 264.7 cells was detected by the MTT assay. RAW 264.7 cells were incubated into 96-well plates at a density of 10000 cells/well and were allowed to adhere overnight. Cells were treated with FZHY in different concentrations (0, 12.5, 25, 50, 100, 200, 400, 800, and 1000 *μ*g/mL) and under 1400W (50 *μ*M), respectively. After 24 hours, 0.5 mg/ml MTT was added to each group and was incubated for additional 4 hours. The supernatant of each group was discarded and then the absorbance at 490 nm with formazan-DSMO dissolution was read. The cell availability of each group was calculated compared to the cell availability of the control group as 100%.

### 2.5. Nitrite Assay

RAW 264.7 cells were seeded into 96-well plates at a density of 100,000 cells/well and were divided into the control group, the model group, FZHY treatment groups (25, 50, 100, and 200 *μ*g/mL), and the positive drug group 1400W (50 *μ*M, a selective inhibitor of iNOS), respectively. After serum-free treatment for 24 h, the control group was treated with serum-free 1640 medium, and the model group was stimulated with LPS (100 ng/mL)/IFN-*γ* (100 U/mL), while the FZHY administration groups were treated with 25, 50, 100, and 200 *μ*g/mL and stimulators LPS (100 ng/mL)/IFN-*γ* (100 U/mL). After 24 hours, 100 *μ*L of the supernatant of each group was collected and added to 100 *μ*L of Griess reaction reagent for 10 mins and then was read at 540 nm using a microplate reader. Then, the nitrite content in the cell supernatant was calculated using the nitrate standard curve.

### 2.6. Animal Experiment

The murine liver fibrosis model induced by CCl_4_ and administration with or without FZHY was proceeded in a previous study [20], and preserved livers were used for further experiments to detect the inflammation-relevant mediators.

### 2.7. qPCR Analysis

RAW 264.7 cells were cultured and divided into the control, model, and FZHY treatment groups (100 and 200 *μ*g/mL), respectively. After being allowed to adhere overnight, cells were treated with serum-free 1640 medium for 24 h. The samples of cells and liver tissues were collected, and the total RNA was extracted by the Trizol method, and miR-155, iNOS, CD86, CD40, IL-6, PPAR-*γ*, and HO-1 were detected. The primers of qRT-PCR for iNOS, CD86, CD40, IL-6, PPAR-*γ*, and HO-1 are listed in Table 1.

### 2.8. Western Blot Analysis

RAW 264.7 cells were cultured in 30 mm culture dishes with 1 × 10^6^ cells. The protein samples of each group were collected, and western blotting was performed after protein denaturation. The protein expressions of iNOS, HO-1, CD40, CD86, and IL-6 compared with *β*-actin and the phosphorylation levels of p38, JNK, ERK, and STAT1 compared with total p38, JNK, ERK, and STAT1 were determined.

### 2.9. Statistical Evaluation

Data were presented as the mean ± SD of results obtained from at least three experiments. Data were assessed by ANOVA analysis and the *t*-test. *P* < 0.05 was considered as statistically significant.

## 3. Results

### 3.1. FZHY Suppressed the Expression of Inducible Inflammatory Synthase iNOS *In Vitro and In Vivo*

**Figure 1:**
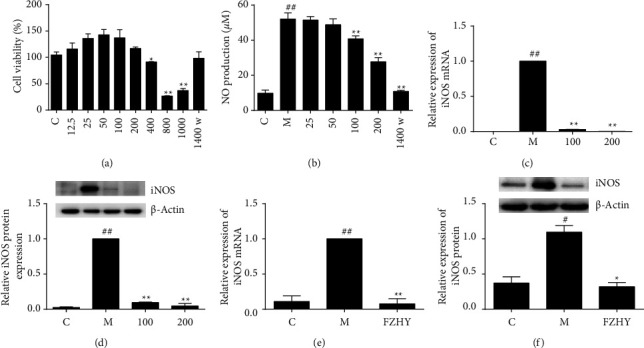
FZHY inhibited iNOS in inflammatory macrophages and mice CCl_4_-treated liver tissues. (a) Cell viability was determined by MTT assay. (b) NO production was evaluated by Griess reaction following incubation with model group (LPS/IFN-*γ*), control group (vehicle control), and 25–200 *μ*g/ml FZHY treatment groups for 24 h. (c) iNOS mRNA in each group from cells was assessed by qPCR. (d) iNOS protein in each group from cells was assessed by western blotting. (e) iNOS mRNAs from liver tissues were assessed by qPCR. (f) iNOS proteins from liver tissues were assessed by western blot. Values are presented as the mean ± SD (standard deviation) from at least three replicates. (C) Vehicle control of macrophages or control group mice; (M) model group (stimulation by LPS at 100 ng/mL and IFN-*γ* at 100 ng/mL or CCl_4_ induced mice); 1400W (an iNOS selective inhibitor at 50 *μ*M); 100: FZHY treatment group at the concentration of 100 *μ*g/mL on macrophages; 200: FZHY treatment group at the concentration of 200 *μ*g/mL on macrophages; FZHY : FZHY treatment group at the dosage of 5.6 g/kg/day for 6-week administration on CCl_4_-induced mice; ##*P* < 0.01 versus C group; #*P* < 0.05 versus C group; ^*∗∗*^*P* < 0.01 versus M group; ^*∗*^*P* < 0.05 versus M group.

To examine the cell viability, RAW 264.7 cells were incubated with FZHY at increasing concentrations (0, 12.5, 25, 50, 100, 200, 400, 800, and 1000 *μ*g/mL) and with 1400W (50 *μ*M) for 24 h. FZHY presented no significant influence on cell viability up to 200 *μ*g/mL by the MTT assay as well as 1400w at the dosage of 50 *μ*M (Figure 1(a)).

iNOS has been used as the major biomarker for the definition of M1 proinflammatory macrophages, so we further examined the effect of FZHY at dosages of 25, 50, 100, and 200 *μ*g/mL on nitrite accumulation, the stable oxidative metabolite of nitric oxide, and in the supernatant from differently treated cells. FZHY inhibited nitrite accumulation induced by LPS plus IFN-*γ* in a concentration-dependent manner (Figure 1(b)). As expected, FZHY inhibited iNOS expressions at gene and protein levels in a concentration-dependent manner on inflammatory macrophages (Figures 1(c) and 1(d)). 1400W, a well-known inducible nitric oxide synthase (iNOS) selective inhibitor, reduced nitrite production by 97.45% at 50 *μ*M compared to the model group induced by LPS plus IFN-*γ*.

Compared with the normal group, the infiltration of inflammatory cells and collagen deposition in the portal area were significantly increased in CCl_4_-induced liver fibrosis mice, while FZHY presented the prevention and curing effects on CCl4-induced liver inflammation and fibrosis [20]. iNOS also altered its expression in liver fibrosis. iNOS deficiency improved liver inflammation and genes encoding collagen, leading to decrease fibrosis [22]. We continued to test iNOS in the liver tissues from CCl_4_-induced liver fibrosis mice with and without FZHY administration. Results demonstrated that FZHY administration did reduce iNOS expression in liver tissues (Figures 1(e) and 1(f)).

Based on these results, FZHY strongly attenuated iNOS in inflammatory macrophages and liver tissues from CCl_4_-induced liver fibrosis mice.

### 3.2. FZHY Enhanced Expression of Anti-Inflammatory Enzyme HO-1

**Figure 2:**
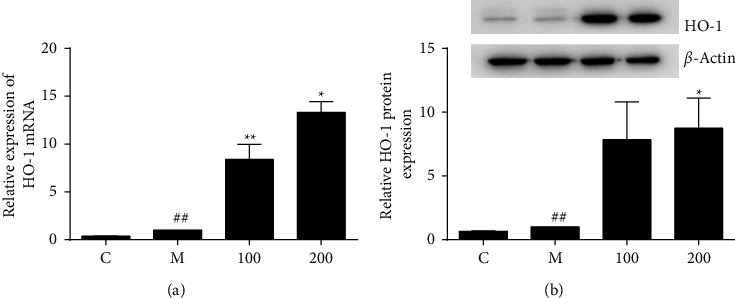
FZHY induced anti-inflammatory HO-1. (a) HO-1 mRNA in each group was assessed by qPCR. (b) HO-1 protein in each group was assessed by western blot. Values are presented as the mean ± SD of at least three replicates. ##*P* < 0.01 versus C group; ^*∗∗*^*P* < 0.01 versus M group; ^*∗*^*P* < 0.05 versus M group.

HO-1 is the inducible and rate-limiting enzyme in heme catabolism and exhibits anti-inflammatory functions to resolve cellular oxidative stress and inflammatory cascade reaction. LPS failed to induce iNOS production in HO-1-overexpressing cells suggesting that HO-1 protected RAW 264.7 cells from inflammation damage [23]. Therefore, we examined the effect of FZHY on HO-1 using qPCR and western blot analysis on macrophages. Results showed that FZHY strikingly increased expressions of HO-1 at gene and protein levels (Figure 2).

### 3.3. FZHY Reduced Other Inflammatory Mediators

**Figure 3:**
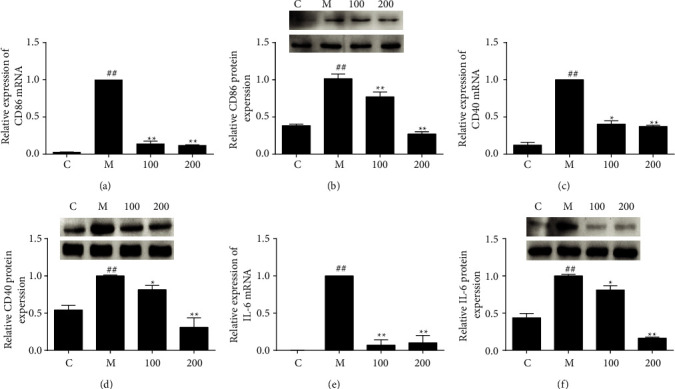
FZHY regulated the proinflammatory mediators. The mRNA and protein levels of markers including CD86 (a, b), CD 40 (c, d), and IL-6 (e, f) were detected by qPCR and western blotting. Values are presented as the mean ± SD of at least three replicates. ##*P* < 0.01 versus C group; ^*∗∗*^*P* < 0.01 versus M group. ^*∗*^*P* < 0.05 versus M group.

iNOS, CD86, CD40, and IL-6 are typical proinflammatory mediators in M1 macrophages [7]. Results showed CD86, CD40, and IL-6 mRNA and protein levels were augmented in LPS plus IFN-*γ* stimulated macrophages compared with the control group. The expressions of inflammatory mediator concentration dependently reduced after FZHY treatment. These findings suggested that FZHY restrained expressions of these proinflammatory mediators (Figure 3).

### 3.4. STAT1/MAPK Signaling Pathways Were Involved in the FZHY-Led Effect

The signal transducer and activator of transcription 1 (STAT1) were involved in the mediation of IFN-*γ* intracellular signaling [24]. Mitogen-activated protein kinases (MAPKs), including p38, extracellular signal-regulated kinase (ERK), and c-JunN-terminal kinases (JNK), played pivotal roles in inflammatory responses [25]. Activated STAT1/MAPK signal pathways led to the induction of iNOS and other proinflammatory cytokines.

**Figure 4:**
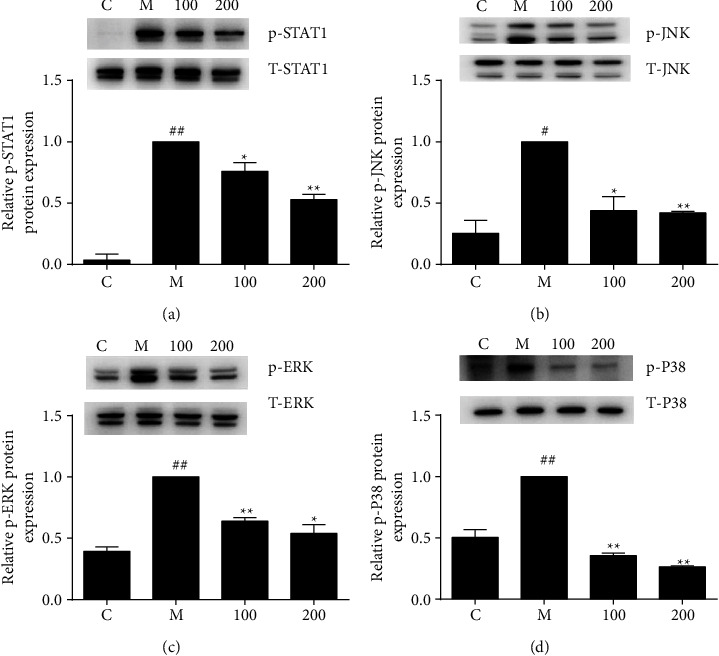
Effects of FZHY on STAT1/MAPKs signaling pathways. (a) p-STAT1 and STAT1, (b) p-JNK and JNK, (c) p-ERK and ERK, and (d) p-p38 and p38 protein expressions were assessed by western blotting and calculated using an imaging system. Values are presented as the mean ± SD of at least three replicates. ##*P* < 0.01 versus C group; #*P* < 0.05 versus C group; ^*∗*^*P* < 0.05 versus M group; ^*∗∗*^*P* < 0.01 versus M group.

Thus, we next investigated whether FZHY inhibited iNOS expression by regulating STAT1/MAPK pathways. LPS plus IFN-*γ* increased the phosphorylation levels of MAPK and STAT-1. FZHY treatment concentration dependently abrogated phosphorylation of STAT1 and MAPK (Figure 4).

These data suggested that STAT1/MAPK pathways were involved in the FZHY-led effect.

### 3.5. FZHY Modulated miR-155/PPAR-*γ* Axis

**Figure 5:**
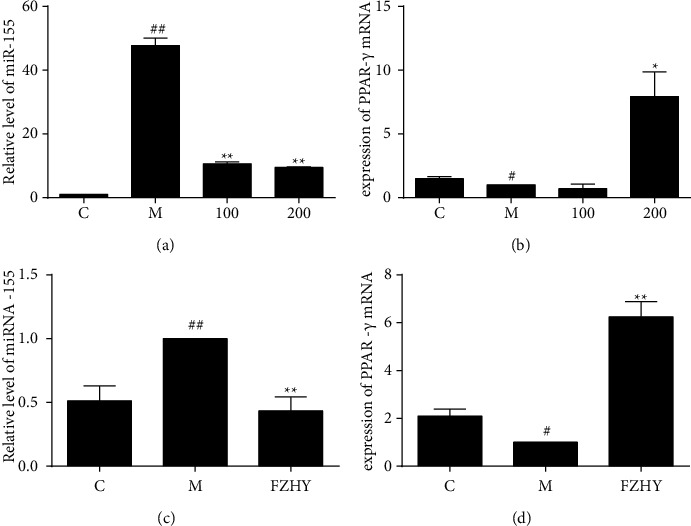
FZHY regulated miR-155/PPAR-*γ* axis. miR-155 and PPAR-*γ* mRNA in each group from macrophages (a, b) and liver tissues (c, d) were assessed by qPCR. Values are presented as the mean ± SD of at least three replicates. ##*P* < 0.01 versus C group; #*P* < 0.05 versus C group; ^*∗∗*^*P* < 0.01 versus M group; ^*∗*^*P* < 0.05 versus M group.

miR-155/PPAR-*γ* axis regulated the progress of inflammation and liver fibrosis [9]. Under the inflammation condition, the level of miR-155 was notably boosted. However, FZHY dramatically struck the elevated level of miR-155 and upregulated expression of its target gene PPAR-*γ* on macrophages and liver tissues from CCl_4_-induced liver fibrosis mice (Figure 5).

### 3.6. Chemical Quality Control of FZHY by HPLC Analysis

**Figure 6:**
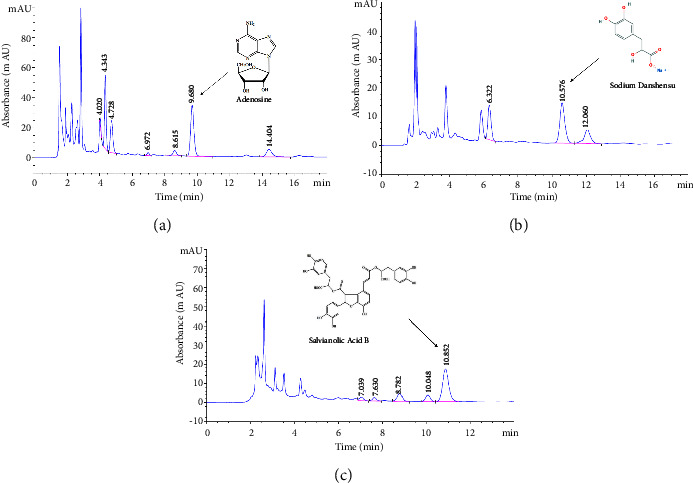
HPLC analysis of FZHY using three ingredients.

We identified three compounds as the chemical quality control of FZHY by HPLC analysis (Figure 6). The content of sodium danshensu (8.3%), salvianolic acid B (13.25%), and adenosine (3.95%) in FZHY met the requirements of Chinese Pharmacopoeia.

## 4. Discussion

Inflammation is a key component and a contributor to profibrogenic progress. Increasing evidence showed that anti-inflammatory therapy exerted its effect in the treatment of liver fibrosis [26]. Targeting chronic inflammation in the context of fibrogenesis might lead to potential antifibrotic therapies. Macrophages play a central role in the progression of liver inflammation and fibrosis progression [27]. M1 macrophages induced enzymes and secreted cytokines to regulate fibrogenesis [28]. Thus, controlling M1 macrophage polarization during fibrosis provides a crucial strategy.

LPS plus IFN-*γ* can activate M1 macrophages that exert a proinflammatory phenotype. iNOS is a significant marker of M1 macrophages. Excessive NO, a gas signal molecule with high reactive properties, produced by iNOS results in oxidative and nitroxidative stress under inflammatory conditions. These findings demonstrated that FZHY suppressed the expression of iNOS at gene and protein levels in a concentration-dependent manner. The accumulation of nitrite and the steady production of NO are also reduced by FZHY (Figure 1). Thus, FZHY's anti-inflammatory activity depends on its inhibition of NO and iNOS. Other M1 markers, including CD86, CD40, and IL-6, were also diminished upon FZHY administration (Figure 3).

Current evidence demonstrated that HO-1 presented a crucial role in anti-inflammatory response and antioxidant progress [29]. Induction of HO-1 in LPS stimulated macrophages and suppressed the production of proinflammatory mediators, while deficiency of HO-1 presented an inflammatory phenotype [30]. Induction of HO-1 caused by the NO produced from iNOS attenuated iNOS expression and NO production [31]. There is a negative feedback loop between HO-1 and iNOS. A previous study showed that FZHY upregulated the antioxidative gene HO-1 in ameliorating nutritional fibrosing steatohepatitis [32]. Notably, FZHY also simultaneously induced antioxidative enzyme HO-1 expression to resolute inflammatory damage triggering a cellular protective mechanism (Figure 2). These data implied that the anti-inflammatory action of FZHY was partially attributed to the induction of HO-1.

The STAT1/MAPK signaling pathways were involved in the progression of inflammation [33]. Results suggested that the activation of STAT1/MAPK pathways was remarkably abolished by FZHY (Figure 4).

microRNAs (miRNAs) are crucial for the progression of inflammation and fibrosis. Accumulating studies suggested that proinflammatory miR-155 promoted liver fibrosis. Elevated miR-155 was observed in a mouse model of liver fibrosis and cirrhotic livers of alcoholic patients. Profibrotic genes by alcohol diet or CCl_4_ treatment were reduced in miR-155 KO mice [9]. miR-155 targeted PPAR-*γ*, SMAD2/5, Snail1, and STAT3 to regulate fibrosis phenotype [34]. miR-155 inhibitor increased the expression of PPAR-*γ* in alcohol-treated macrophages. Activation of PPAR-*γ* suppressed iNOS expression in M1 macrophages and displayed anti-inflammatory properties. The tyrosine nitration of PPAR-*γ* by iNOS impaired its transcriptional activity and stability [35].

A network pharmacology approach and a cell-based assay revealed that schisandrin B, salvianolic acid A, and kaempferol from FZHY could bind to PPAR-*γ* [19]. We found that proinflammatory miR-155 increased while its target gene PPAR-*γ* decreased upon stimulation by LPS in IFN-*γ* or CCl_4_-induced liver fibrosis mice, while FZHY reduced the level of miR-155 and upregulated the expression of PPAR-*γ* (Figure 5).

## 5. Conclusions

**Figure 7:**
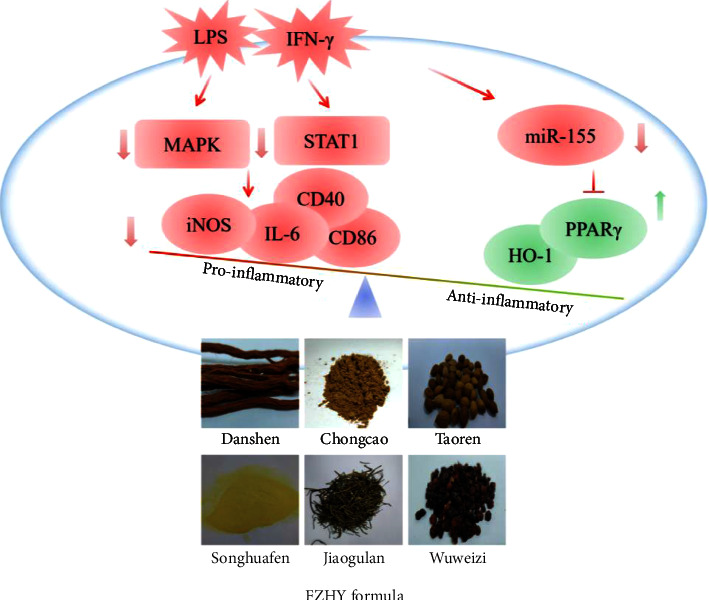
Anti-inflammatory mechanisms summary of FZHY.

Collectively, our findings demonstrated that FZHY exerted an anti-inflammatory effect on LPS plus IFN-*γ* -induced inflammation with modulation of proinflammatory and anti-inflammatory mediators via MAPK/STAT-1 signaling pathways and miR-155/PPAR-*γ* axis (Figure 7). Future studies are needed to elucidate active compounds from FZHY and core targets and pathways in depth underlying the relationship between inflammation and fibrosis.

## Figures and Tables

**Table 1:** Primer sequences used for qRT-PCR amplification.

Gene	Sense sequence	Antisense sequence
iNOS	5′-GGAGCGAGTTGTGGATTGTC-3′	5′-GTGAGGGCTTGGCTGAGTGAG-3′
CD86	5′-GCACGGACTTGAACAACCAG-3′	5′-CCTTTGTAAATGGGCACGGC-3′
CD40	5′-ATTTGTGCCAGCCAGGAAGCCG-3′	5′-GCATCCGGGACTTTAAACCACAGA-3′
IL-6	5′-CCACTTCACAAGTCGGAGGCTTA-3′	5′-GTGCATCATCGCTGTTCATACAATC-3′
PPAR-*γ*	5′-AGACCACTCGCATTCCTTTGAG-3′	5′-GCAGGTTCTACTTTGATCGCACT-3′
HO-1	5′-CACAGATGGCGTCACTTCGTC-3′	5′-GTGAGGACCCACTGGAGGAG-3′
GAPDH	5′-AACGGATTTGGTCGTATTGGG-3′	5′-CAGGGGTGCTAAGCAGTTGG-3′

## Data Availability

The data used to support the findings of this study are included in the article.
